# *Burkholderia stabilis* infections associated with contamination of non-sterile alcohol-free skin cleansing wipes, United Kingdom, 2018 to 2026

**DOI:** 10.2807/1560-7917.ES.2026.31.9.2600145

**Published:** 2026-03-05

**Authors:** Aideen Carroll, Rebecca Stretch, Dan Blackman, Ashley Popay, Dervla Kenna, Michaela Day, Caroline Willis, Karren Staniforth, David Williams, Karen Osman, Georgina Russell, Jennie Papprill, Jack Gordon-Brown, Gemma Fear, Mariyam Mirfenderesky, Dakshika Jeyaratnam, Catherine Searle, Richard Pebody, Colin S Brown, Sarah L Milligan, James Elston

**Affiliations:** 1 United Kingdom Health Security Agency (UKHSA), Colindale, United Kingdom; 2 Department of Health & Social Care, London, United Kingdom; 3 National Institute for Health Research Health Protection Research Unit (NIHR HPRU) in Healthcare Associated Infections and Antimicrobial Resistance, University of Oxford, Oxford, United Kingdom

**Keywords:** epidemiology, outbreaks, bacterial infections

## Abstract

A *Burkholderia stabilis* (ST480) outbreak associated with skin cleansing wipes has comprised 59 confirmed cases in the United Kingdom 2018–2026. Cases included patients with co-morbidities and clinically relevant infections. There was one associated death. *Burkholderia stabilis* was recovered from non-sterile alcohol-free cleansing wipes which did not have the relevant medicines authorisation. Products were suspended from sale though not recalled, and the outbreak continued following public health intervention. We highlight risks of potential relevance to other countries.

Medical and personal care product contamination with *Burkholderia cepacia* complex (Bcc) and associated outbreaks have been reported internationally, and in the United Kingdom (UK) [1-6]. *Burkholderia stabilis* is a member of the Bcc, intrinsically resistant to several antibiotics and can cause considerable morbidity and mortality in people with chronic diseases such as cystic fibrosis (CF) [7]. Here we describe an ongoing outbreak of *B. stabilis* associated with non-sterile alcohol-free cleansing wipes, resulting in clinical infections and one death. We highlight implications and potential risks relevant to international partners.

## Outbreak detection and investigation

In November 2021, a cluster of 18 cases of an uncommon sequence type of *B. stabilis* was identified by the UK Health Security Agency (UKHSA) Antimicrobial Resistance and Healthcare Associated Infections (AMRHAI) reference unit. These cases were geographically disseminated with specimens submitted from acute hospital settings. An investigation was initiated, suspecting a common source product exposure. Case definitions were developed (Box). Confirmed cases were defined by whole genome sequencing (WGS); with probable cases identified through interrogation of national surveillance data (Second Generation Surveillance System) [8].

Box **Definitions for confirmed and probable cases, *Burkholderia stabilis* ST480 outbreak, United Kingdom, 2018–2026**

**Probable case:**

• Any person in the UK with a local laboratory-confirmed isolate of *Burkholderia stabilis* of unknown ST

OR

• Any person in the UK with a local laboratory-confirmed isolate of *Burkholderia* spp., regardless of species or ST AND with an epidemiological link to another probable or confirmed case.

**Confirmed case:**

• Any person in the UK with a reference laboratory confirmed isolate of *Burkholderia stabilis* ST480.

ST: sequence type; UK: United Kingdom.

Information was obtained through virtual meetings with clinical and infection prevention and control (IPC) teams or cases, and via a trawling questionnaire. The questionnaire was designed to capture information on cases (including demographics, co-morbidities), clinical illness, and relevant exposures; and was shared by the investigation team for completion by IPC teams. Product sampling was undertaken based on exposures of interest identified by clinical teams, and assessment of biological plausibility as vehicle for Bcc contamination (prioritising non-sterile aqueous-based products). Products were purposively selected and submitted to the UKHSA Food, Water and Environmental (FWE) Microbiology laboratory for testing using methodology previously outlined [2]. Product testing was conducted from investigation onset. The investigation has been ongoing from 2021 to 2026 inclusive; whilst the product source was identified in 2025, cases continue to be followed up to identify and mitigate potential associated risks.

## Genotyping

Genotyping of Bcc isolates (retrieved from cases and products) was performed at AMRHAI using *recA* sequencing. Isolates underwent WGS at the UKHSA Colindale Sequencing Laboratory using the Illumina NextSeq platform (Illumina, San Diego, the United States (US)), following library preparation using Illumina DNA Prep. Isolate sequence read datasets that passed a quality filtering stage were retained for inclusion into a multiple alignment by split-*k-mer* analysis. After further quality filtering of the alignment, a phylogenetic reconstruction was compared with the expectation of low diversity from a common source.

## Case characteristics

**Figure 1:**
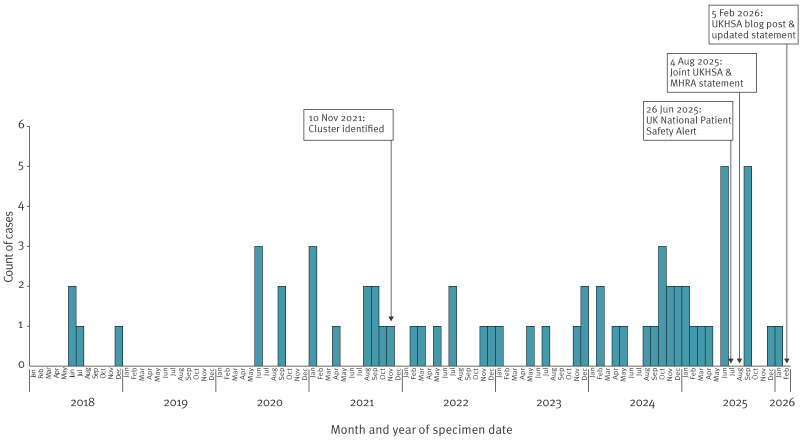
Temporal distribution of cases, *Burkholderia stabilis* ST480 outbreak with dates of key interventions, United Kingdom, 1 June 2018–8 January 2026 (n = 62)

MHRA: Medicines & Healthcare products Regulatory Agency; ST: sequence type; UK: United Kingdom: UKHSA: UK Health Security Agency.

As of February 2026, 59 confirmed cases and three probable cases have been identified (Table). Cases comprised individuals across a wide age range (0–93 years, median: 44 years), of which 32 (54%) were females and 27 (46%) were males. Specimen dates were between 11 June 2018 and 8 January 2026 (Figure 1) and specimen types included blood (39/62; 63%), wound or skin swabs (16/62; 26%), line tip (2/62; 3%), bone/tissue (3/62; 5%), cerebrospinal fluid (1/62; 2%) and joint fluid (1/62; 2%). Specimens were predominantly retrieved from sterile sites (45/62; 73%). Clinical teams reported clinically relevant Bcc infections in 25 of 34 cases where information was available (Table).

**Table:** Characteristics of confirmed and probable cases, *Burkholderia stabilis* ST480 outbreak, United Kingdom, June 2018–January 2026 (n = 62)

Characteristic	Confirmed (n = 59)	Probable (n = 3)
Age category (years)		
0–19	15	1
20–39	12	0
40–59	14	0
≥ 60	18	2
Sex		
Male	27	2
Female	32	1
Specimen type		
Invasive^a^	43	2
Non-invasive	16	1
Clinically relevant infection^b^		
Yes	25	0
No	9	0
Unknown	25	3
Indwelling intravascular devices (n = 50)^c^		
Yes	37	0
No	13	0
Weakened immune systems (n = 32)^c^		
Yes	13	0
No	19	0
Co-morbidities (n = 50)^d^		
Malignancy	19	0
Chronic respiratory disease	4	0
Diabetes	7	0
Cystic fibrosis	0	0

Bcc: *Burkholderia cepacia* complex; CSF: cerebrospinal fluid; ST: sequence type.

^a^ Isolation of the organism from blood culture, CSF or other normally sterile body site.

^b^ As reported by clinician or where Bcc infection was treated with an antibiotic regimen.

^c^ Information extracted from trawling questionnaires and clinical follow-up.

^d^ Information as reported by clinician, available for 32 cases.

Cases were commonly reported to have a co-morbid illness; including 19 of 50 with malignancy and 13 of 32 with weakened immune systems (where information was available). No cases were known to have cystic fibrosis (CF). Five confirmed and one probable case died within 30 days of detection of *B. stabilis*. One death was attributed to their *Burkholderia* infection.

Cases were identified from all nine UKHSA regions of England and from Scotland, Wales and Northern Ireland. There were three instances of cases sharing a common healthcare provider or services. For cases admitted to acute hospitals, specimens from 22 of 31 cases (where information available) were collected within 48 h of admission, suggesting community exposure. However, cases often had preceding complex care input across multiple settings, which made the exposure setting challenging to ascertain.

Thirty-seven of 50 cases (where information available) had indwelling intravascular devices or recent line insertion/removals, of which 31 were central lines. *Burkholderia* was detected in blood or line tip specimens from 34 of these cases. Of the 14 remaining cases, 11 were reported to have had a breach of skin integrity (e.g. wounds).

Information was available for 12 of 15 paediatric cases: 10 were being treated for long-term conditions (including malignancy), nine of which had longstanding intravenous lines in situ. In these 10 cases, *B. stabilis* was recovered from blood specimens collected around the time of admission, suggesting exposure before hospitalisation.

## Product investigations and genotyping results

We tested 187 products at the FWE laboratory from 2022 to February 2026 (inclusive). In June 2025, *Burkholderia* spp. was recovered from a skin cleansing wipe apparently intended for first aid. This product, submitted by a hospital team, had been purchased by the case for home use. Further unopened stock of the same wipe and other similar wipe brands were tested. *Burkholderia* spp. contamination was identified in four product brands.

**Figure 2:**
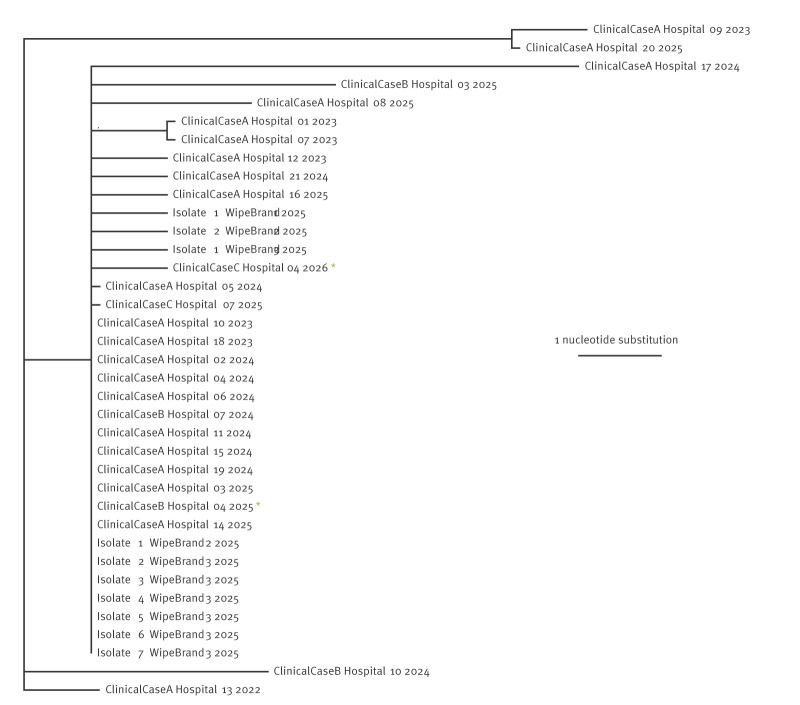
Phylogenetic reconstruction from 5,105,640 positions of aligned whole genome sequence of *Burkholderia stabilis* ST480 isolates from clinical cases (n = 27) and product packets (n = 10), United Kingdom, 2022–2026

Isolates from three of the four products were identified as *B. stabilis* ST480. These originated from the same UK manufacturing site. Phylogenetic analysis (Figure 2) highlighted that there was minimal or no diversity observed between genome sequences of clinical *B. stabilis* ST480 isolates and those from products, supporting a common source hypothesis. Of note, ClinicalCaseC Hospital07 2025 had an identified exposure to the product Isolate1 WipeBrand3 2025. *Burkholderia* spp. isolated from the remaining product (produced by a different manufacturer) was identified as *Burkholderia aenigmatica* ST855, which had not previously been identified in the UK by AMRHAI.

ST: sequence type.

Cases marked with a green asterisk had specimen dates after August 2025 (e.g. after initial public health intervention).

## Public health measures

Upon identification of the contaminated product, an Incident Management Team led by UKHSA was established, with representation from stakeholders, including the UK medicines regulator (Medicines & Healthcare products Regulatory Agency (MHRA)), Department of Health and Social Care, health system procurement colleagues, and public health institutions of Scotland, Wales and Northern Ireland.

The contaminated products identified were found to be widely available for public purchase online and commonly included in first aid kits in the UK. These products were not procured by the national health system supply chain.

The MHRA determined the products to be medicinal products under the definition set out in the Human Medicines Regulations (HMRs) 2012 [9] but they did not hold relevant MHRA authorisations. Working with the manufacturer and distributors, sale and supply of the contaminated wipes was ceased. The products could not be recalled by MHRA as they were not authorised products and sat outside of the legal UK medicines supply chain. Products from which *B. stabilis* ST480 was recovered were not known to be distributed outside of the UK and Ireland. However, the product from which *B. aenigmatica* ST855 was recovered was additionally distributed in two countries outside of the UK.

In June 2025, communications were issued to the UK health system, including via a National Patient Safety Alert [10]. International partners were informed and updated (e.g. via the National International Health Regulations Focal Points). A joint UKHSA and MHRA communication was released in August 2025 to inform the UK public, listing contaminated products and reinforcing appropriate intravascular device care and first aid wound care [11]. Following public health intervention, cases continued to be identified. There were seven confirmed cases with specimen dates between September 2025 and January 2026. *Burkholderia* was recorded as the primary cause of death for the most recent case.

Due to continuing case ascertainment and associated death, UKHSA re-established a multi-agency incident response in January 2026. Further risk communications included an alert to the health system and an updated press release. These reiterated guidance on appropriate use of (sterile) wipes for wound care and good infection prevention and control (IPC) practice for care of intravascular devices [11,12].

## Discussion

*Burkholderia stabilis* is intrinsically resistant to several antibiotics and can cause considerable morbidity and mortality in people with chronic diseases such as CF [7]. Medical and personal care product contamination with Bcc has been previously reported [1-6].

Identifying the contaminated product in this outbreak was challenging. As specimens were mainly collected in inpatient settings, trawling questionnaires were predominantly completed by staff with limited information about products used outside of hospital. Community healthcare providers and cases were contacted where appropriate. However, time lag between specimen collection and request for exposure information potentially impacted memory recall of exposures. Characteristics of identified cases were also unlikely to be representative of the wider exposed population, given enhanced surveillance only extracted reports of invasive specimens from sterile sites and specimens submitted by healthcare workers may have been more likely to be from clinically relevant infections.

Sequencing and phylogenetic analysis were critical to identification of a common source, with minimal diversity noted between product and case isolates despite wide geographical and temporal dissemination of cases.

Once contamination was identified, product sale and supply were ceased under the direction of the MHRA. Products were not authorised as medicines and therefore should not have been available for sale as marketed, as quality could not be assured. However, recall was not mandated, meaning that products already sold or in the supply chain remained available. The hypothesis, which continues to be supported by phylogenetic analysis, is that residual products (available individually or as part of first-aid kits) remain the source of infection.

We assume that affected wipes were typically used in the community; with invasive infections potentially arising from application to broken skin, wounds and/or contact with intravenous line ports (either directly or indirectly via contamination of hands or surrounding skin).

Products involved were marketed but not authorised as medical products, and therefore without assurance of safety and quality. In the absence of product recall, UK public health interventions have focused on communicating safe IPC practice in intravascular line care and ensuring sterile wipes are used for wound care (reinforcing that alcohol-free non-sterile wipes should not be used for these purposes). Healthcare professionals and the public have also been advised to discard affected wipes. Messaging required recent reinforcement due to further cases, potentially highlighting the limitations of public health communications as a standalone intervention.

## Conclusion

The *B. stabilis* ST480 outbreak is ongoing and associated with non-sterile alcohol-free skin cleansing wipes. These products were previously widely available for sale within the UK, though risk associated with this outbreak may extend internationally. Our experience highlights potential risk of non-sterile products that may be used for healthcare purposes, and ongoing need to enhance their safety in partnership with regulators, industry, and healthcare and public health institutions globally. Given the predilection of the Bcc to cause product contamination events resulting in patient harm, we advocate for enhanced Bcc surveillance and timely outbreak response.

## Data Availability

The whole genome sequencing read data for *Burkholderia stabilis* ST480 isolates included in the phylogenetic analysis have been deposited in the European Nucleotide Archive as Bioproject PRJEB105547.
